# The Behaviour of Bifilm Defects in Cast Al-7Si-Mg Alloy

**DOI:** 10.1371/journal.pone.0160633

**Published:** 2016-08-16

**Authors:** Mahmoud Ahmed El-Sayed

**Affiliations:** Department of Industrial and Management Engineering, Arab Academy for Science, Technology and Maritime Transport Abu Qir, Alexandria, Egypt; VIT University, INDIA

## Abstract

Double oxide films (bifilms) are significant defects in the casting of light alloys, and have been shown to decrease tensile and fatigue properties, and also to increase their scatter, making casting properties unreproducible and unreliable. A bifilm consists of doubled-over oxide films containing a gas-filled crevice and is formed due to surface turbulence of the liquid metal during handling and/or pouring. Previous studies has shown that the nature of oxide film defects may change with time, as the atmosphere inside the bifilm could be consumed by reaction with the surrounding melt, which may enhance the mechanical properties of Al alloy castings. As a proxy for a bifilm, an air bubble was trapped within an Al-7wt.%Si-0.3wt.%Mg (2L99) alloy melt, subjected to stirring. The effect of different parameters such as the holding time, stirring velocity and melt temperature on the change in gas composition of the bubble was investigated, using a design of experiments (DoE) approach. Also, the solid species inside the bubbles solidified in the melt were examined using SEM. The results suggested that both oxygen and nitrogen inside the bifilm would be consumed by reaction with the surrounding melt producing MgAl_2_O_4_ and AlN, respectively. Also, hydrogen was suggested to consistently diffuse into the defect. The reaction rates and the rate of H diffusion were shown to increase upon increasing the holding time and temperature, and stirring velocity. Such significant effect of the process parameters studied on the gaseous content of the bubble suggesting that a careful control of such parameters might lead to the deactivation of bifilm defects, or at least elimination of their deteriorous effect in light alloy castings.

## Introduction

In response to the raising consumer demands for increased performance and fuel economy, the use of aluminium to replace heavier materials (steel or copper) in the automotive and aerospace industries was a justified choice. Aluminium has unique characteristics, such as high elasticity, high formability, relatively low melting point and high electrical and thermal conductivity. Also, many Al alloys have high strength to weight ratio [1,2,3]. Such properties made it very suitable for casting and today, significant amounts of cast aluminium alloys are being used to fabricate components such as engine blocks, cylinder heads, wheels, and pistons. The mechanical properties of Al castings were greatly affected by their inclusion contents, particularly double-oxide film defects, or bifilms, which was first introduced by John Campbell since the early nineties [4]. These defects are created due to surface turbulence of the liquid Al, which is a common foundry practice during the metal pouring. If liquid aluminium entered a mould cavity with a velocity greater than a critical value (about 0.5 m/s for most liquid metals [5]), the surface oxide film of the liquid metal would fold over onto itself and be submerged into the bulk liquid with a volume of air entrapped within it [2]. Oxide films were shown to constitute cracks in the solidified casting, which has been found to have detrimental effects on the tensile and fatigue properties of the castings produced. Submerged oxide films were also recognized to initiate hydrogen porosity [6,7], and to act as a nucleation site for Fe-rich intermetallics [8].

In an earlier research [9], it was indicated that the internal atmosphere of bifilms would react with the surrounding liquid Al, and was gradually consumed over time. The results by Nyahumwa et al. [9], Raiszadeh and Griffiths [10], El-Sayed et al. [11,12], and Griffiths et al. [13,14] demonstrated that oxygen within the defect would be consumed first forming Al_2_O_3_, MgAl_2_O_4_ or MgO, depending on alloy composition, followed by nitrogen consumption to form AlN. Also, hydrogen was found to diffuse into the bifilm as the melt was kept longer in the liquid state [15,16]. The consumption of the bifilm interior atmosphere as well as the H ingress into the defect was suggested by El-Sayed et al. to result in an alteration of the shape and size of the bifilm [17,18]. This change in morphology with time suggested that the defect could perhaps become less detrimental to mechanical properties.

The aim of this work was to study the change in the gas content of an air bubble, as an analog for a bifilm defect, using a mass spectrometer. The response surface method, which is a statistical experimental design procedure, was used to investigate the influence of different parameters such as the bubble age, bubble velocity and melt temperature on the reaction rates of O and N inside the air bubble as well as the rate of H diffusion into the bubble. The parameters were also optimised to minimise to amounts of O, N and H within the bubble.

## Experimental

**Fig 1:**
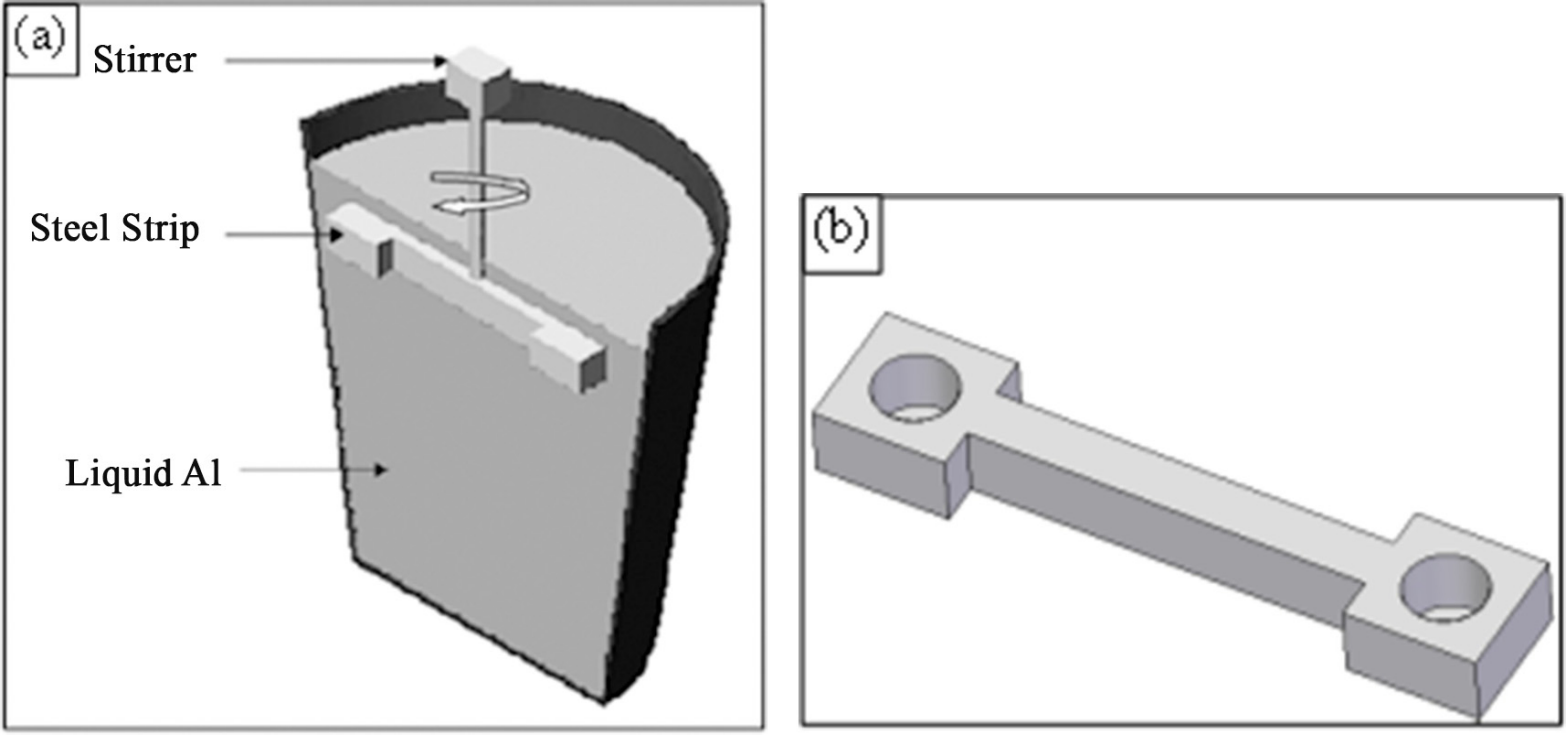
(a) Schematic illustration of the experimental technique, (b) 3D view of the steel strip.

The experimental setup is described in more details elsewhere [11]. In brief, a set of experiments was carried out to analyse the change in gas composition of the internal atmosphere of an air bubble (as a proxy for a bifilm defect) held within an aluminium melt. A stirrer was welded to a steel bar in which a blind hole, of 6 mm dimeter and 5 mm depth, was drilled. About 0.5 kg of 2L99 alloy (Al–7Si–0.3Mg) was melted in a resistance-heated furnace. The steel strip was then immersed into the melt (at a given temperature) to a depth of about 5 mm below the surface of the melt (to avoid surface turbulence during its rotation), and stirred with a given speed for a given period of time, as shown in Fig 1. This was to bring about the air bubble inside the hole in direct contact with the molten metal, which would permit the air inside the bubble to react with the adjacent liquid Al, and hydrogen from the melt to diffuse from/into the air bubble. After a preset holding time the rotation was halted and the melt was allowed to solidify, trapping the air bubble and its contents.

In this study a statistical design of experiment (DoE) using a response surface technique was adapted. In order to determine the equations of the response surface, the central composite second order design was used to approximate the equation using the smallest number of experiments possible. An experimental design was generated to determine the relationship between factors affecting a process and the output of that process and consequently manage these factors to optimise the output. The expression for the second order central composite design is given in Eq 1:

$$Y=b_{o}+\sum b_{i}x_{i}+\sum b_{ii}x_{i}^{2}+\sum b_{ij}x_{i}x_{j}$$ (1)

where Y is process yield (or the response surface), while *x_i_* are the factors or process parameters. The expression contains linear terms in *x_i_*, quadratic terms in $x_{i}^{2}$, and product terms in *x_i_x_j_*. The terms b_i_, b_ii_, and b_ij_ are constant coefficients. Method of least squares is used to determine the constant coefficients. In this way the response surface methodology can quantify the relationship between controllable input parameters and the obtained response surface.

The key process parameters in this study were the holding time (bubble age), stirring speed (bubble velocity) and melt temperature. Table 1 shows the range and levels of the investigated parameters. Also, the output response was the amount of hydrogen, oxygen and nitrogen inside the bubbles. The experimental design matrix was then developed and the experiments were carried out to record the output responses. The results would be used to establish a mathematical model correlating different process parameters to the output response, and afterwards optimising that model using genetic algorithm.

**Table 1:** The range of matrix building parameters.

Parameter	Units	Levels		
		-1	0	1
Bubble Age	min	2	17	32
Bubble Velocity	m/s	0.3	0.85	1.4
Melt Temperature	°C	720	820	920

To perform the DoE and parametric optimisation, 17 parametric combinations were used to fabricate samples using a fractional factorial DoE. As shown in Table 1, three different rotational speeds of the bubble were studied (115, 325 and 540 rpm), corresponding to angular velocities of 0.3, 0.85 and 1.4 ms^-1^, respectively. Also, three different holding times and melt temperatures were considered in this study. The parametric combinations are presented in Table 2.

**Table 2:** Matrix building parameters and the amount of different gases within the bubbles.

Run	Bubble Age (minute)	Bubble Velocity (ms^-1^)	Melt Temperature (°C)	Amount of Oxygen (μmol)	Amount of Nitrogen (μmol)	Amount of Hydrogen (μmol)
1	32	0.85	820	0.01	1.08	0.25
2	17	1.4	820	0.01	1.18	0.17
3	17	0.85	820	0.01	1.25	0.15
4	2	1.4	920	0.08	1.19	0.05
5	32	0.3	720	0.01	1.3	0.13
6	32	0.3	920	0.01	1.05	0.22
7	2	0.3	720	0.3	1.51	0.02
8	32	1.4	720	0.01	1.15	0.23
9	2	0.3	920	0.25	1.4	0.03
10	2	0.85	820	0.19	1.38	0.03
11	17	0.85	820	0.01	1.23	0.16
12	17	0.85	720	0.02	1.39	0.12
13	17	0.85	820	0.01	1.27	0.14
14	2	1.4	720	0.2	1.51	0.02
15	32	1.4	920	0.01	0.76	0.32
16	17	0.3	820	0.04	1.41	0.1
17	17	0.85	920	0.02	1.2	0.16

After solidification, the gaseous content of the bubbles was analysed using a Pore Gas Analyser, (constructed by Hyden Ltd.) containing a mass spectrometer to determine the amounts of hydrogen, oxygen and nitrogen inside the bubbles. Finally, the sample interior was cut out and investigated using SEM to determine the presence of different species such as Al_2_O_3_, MgAl_2_O_4_, MgO, AlN and Mg_2_N_3_. Also, a reference air bubble, of the same dimensions was made by soldering and sealing the two sides of a copper tube of 6 mm dimeter and 5 mm length. This was also analysed so as to provide a calibration sample that should contain the normal composition of the atmosphere.

The original volume of the air bubble was supposed to be 141.4 mm^3^, the same as the hole in the steel bar. The initial volume of oxygen would be 29.7 mm^3^, and the initial mass was 4.1x10^-7^ mol. Similarly, the initial volume and mass of nitrogen inside the bubble would be 110.2 mm^3^ and 1.52x10^-6^ mol, respectively. The results from the Pore Gas Analyse were presented as plots of the change in pressure of each gas in the bubble against time. The area under the pressure-time curve for each gas was integrated to obtain the amount of such gas in the bubble. The values of the area under curve of the oxygen and nitrogen inside the reference sample were compared to the expected masses of both gases in such sample. In this way the amounts of different gases inside the bubbles produced in different experiments were estimated. Further explanation of the analysis of the change of the gaseous content of the bubble could be found in [11].

## Results and Discussion

The analysis of the reference air bubbles detected about 76.5 vol% N, 20.6 vol% O, 0.8 vol% Ar, 1.5 vol% H, and 0.6 vol% water vapor, which were close to the nominal values of the gases in air. The relatively high percentages of hydrogen and water vapor recorded might be resulted from a slight contamination of the environment inside the Pore Gas Analyser. This revealed the ability of the instrument to accurately analyse the contents of the gas bubbles trapped inside the steel holder during the stirring experiments, and showed the reliability of the results obtained with the equipment. The change in the amount of oxygen, nitrogen and hydrogen within bubbles held in 2L99 melts at different temperatures, for different times and subjected to different stirring velocities along with the parametric combinations, are shown in Table 2 above.

The response surface for the amount of each of the hydrogen and nitrogen inside the bubble were suggested to be represented as a two-factor interaction (2FI) model of the parameters of the holding process (i.e. bubble age (A), bubble velocity (V) and melt temperature (T)), that could be given as shown in Eq 2. In addition, the amount oxygen inside the bubble was found to be a quadratic function of the three parameters, which could be expressed as in Eq 3.

$$Response=b_{o}+b_{1}(A)+b_{2}(V)+b_{3}(T)+b_{4}(AV)+b_{5}(AT)+b_{6}(VT)$$ (2)

$$Response=b_{o}+b_{1}(A)+b_{2}(V)+b_{3}(T)+b_{4}(AV)+b_{5}(AT)+b_{6}(VT)+b_{7}{\left(A\right)}^{2}+b_{8}{\left(V\right)}^{2}+b_{9}{\left(T\right)}^{2}$$ (3)

where b_o_ is the average response, and b1, b2,…,b9 are the model coefficients that depend on the main and interaction effects of the holding process parameters. Least Squares Fitting, which is a mathematical procedure for finding the best-fitting curve to a given set of points by minimizing the sum of the squares of the offsets of the points from the curve, was applied to analyse the data presented in Table 2 and to determine the constant coefficients. The values of the coefficients for the response surface of the amount of each of the oxygen, nitrogen and hydrogen inside the bubble are shown in Table 3. The coefficient of correlation (R^2^) of the models describing the relationship between the process parameters and the amount of the oxygen, nitrogen and hydrogen inside the bubble were 0.97, 0.95 and 0.98, respectively. The ANOVA indicated that, within the investigated range of parameters, the most significant factors influencing the amount of both oxygen and hydrogen inside the bubble were the bubble age, bubble velocity and the interaction between both factors, as well as the melt temperature and the interaction between the bubble age and melt temperature. On the other hand, the amount of nitrogen was mainly affected by the bubble velocity and melt temperature along with their interaction, and the bubble age.

**Table 3:** Response surface model coefficients for the amount of oxygen, nitrogen and hydrogen inside the bubble.

Coefficient	Model for the Amount of Hydrogen	Model for the Amount of Nitrogen	Model for the Amount of Oxygen
b_o_	0.14	1.25	0.014
b_1_	0.100	-0.16	-0.097
b_2_	0.029	-0.088	-0.030
b_3_	0.026	-0.13	-0.017
b_4_	0.022	-0.029	0.034
b_5_	0.018	-0.026	0.021
b_6_	2.500E-003	-0.044	-8.750E-003
b_7_	0	0	0.084
b_8_	0	0	8.592E-003
b_9_	0	0	3.592E-003

**Fig 2:**
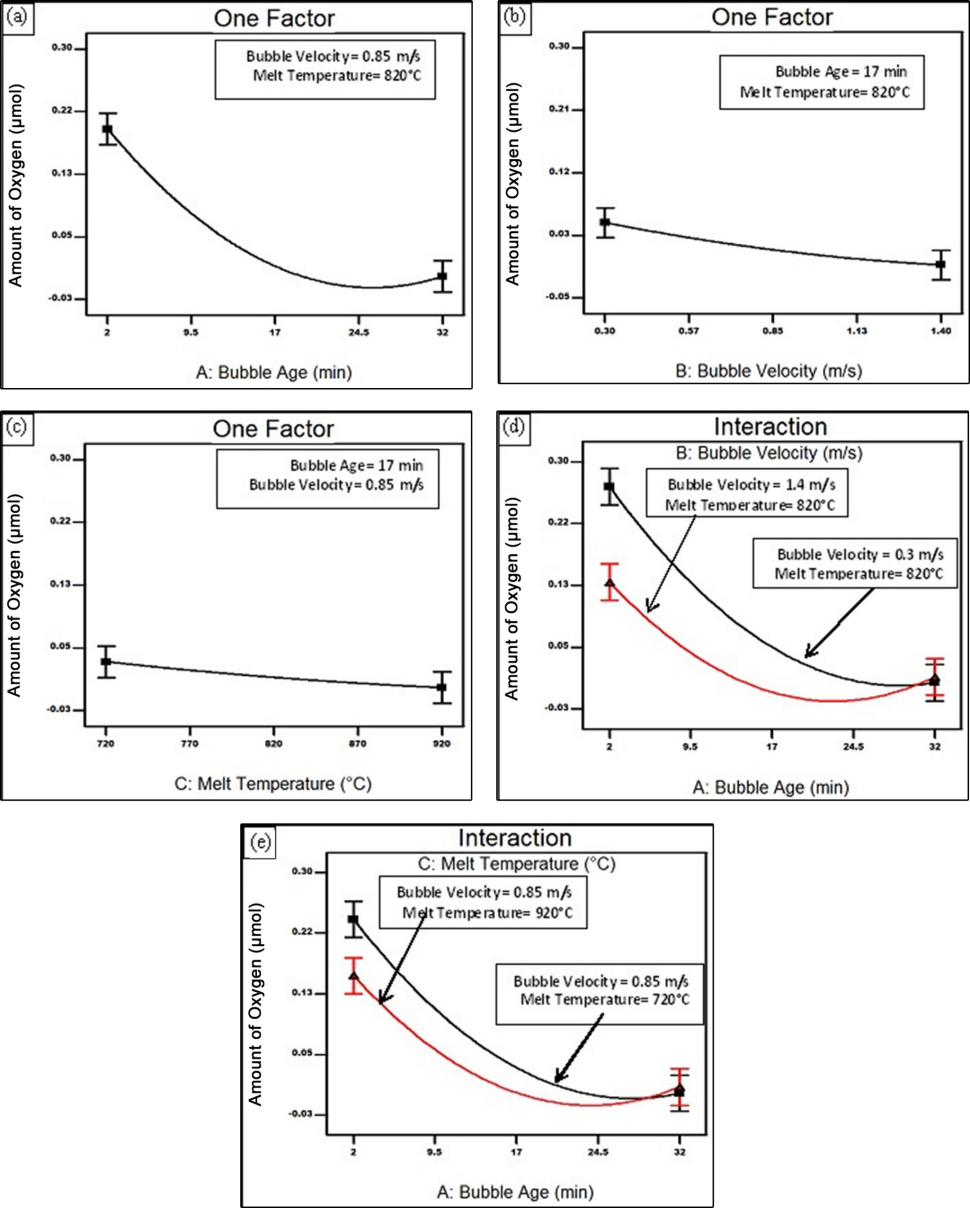
Response surface plot showing the effect of (a) bubble age, (b) bubble velocity, (c) melt temperature, (d) the interaction between bubble age and bubble velocity (e) the interaction between bubble age and melt temperature on the amount of oxygen inside the bubble.

Fig 2A, 2B and 2C show the response surface model prediction of the amount of oxygen inside the bubble with respect to bubble age, bubble velocity and melt temperature. It was shown that the amount of oxygen inside the bubble decreased consistently with increasing either the bubble age, bubble velocity and/or melt temperature. In addition, and as indicated by the model it seems that the interactions between the bubble age and both of the bubble velocity and melt temperature are also significant, as shown in Fig 2D and 2E. At higher bubble velocity or melt temperature the effect of bubble age on the consumption of O from inside the bubble is less considerable.

**Fig 3:**
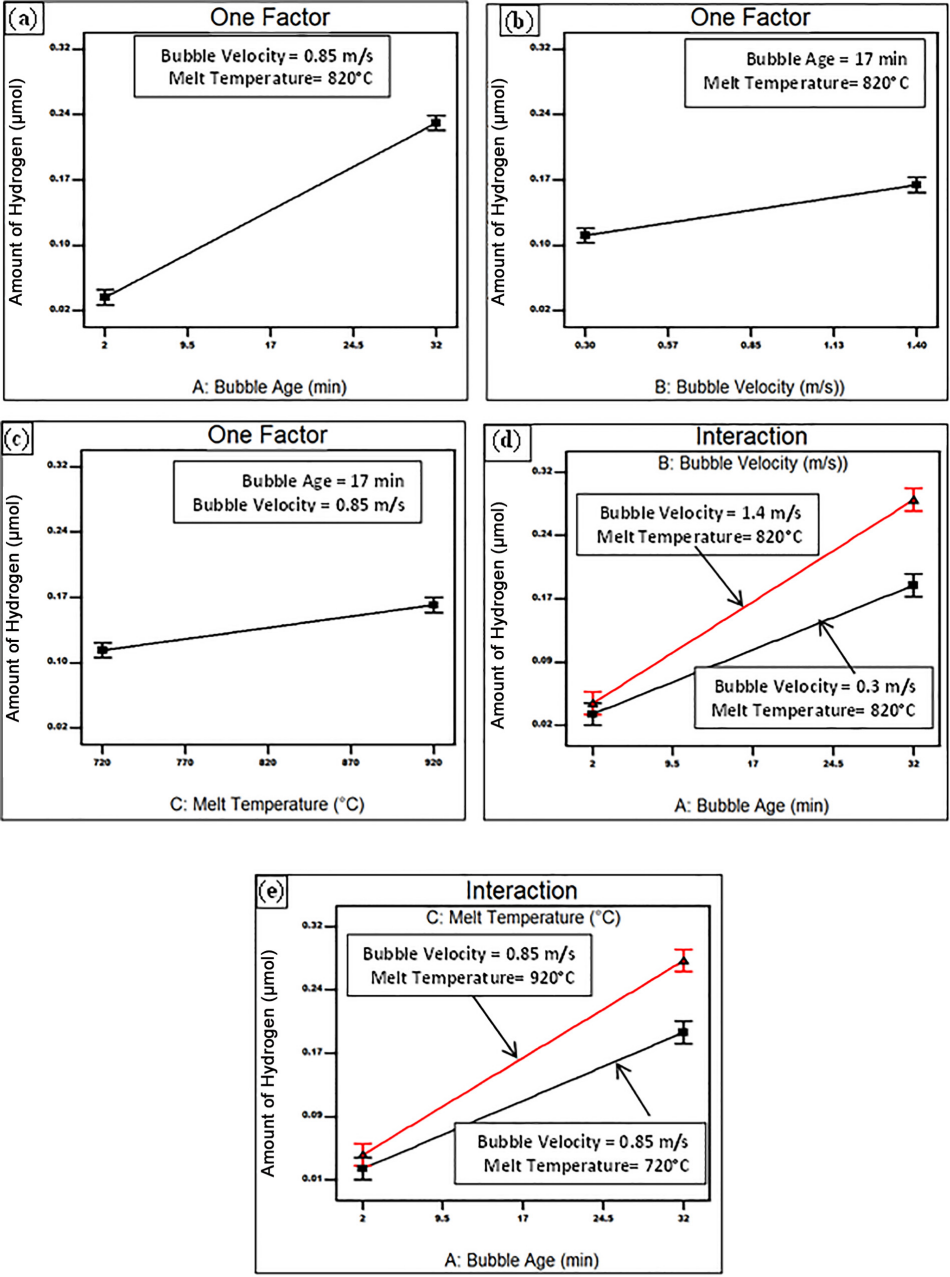
Response surface plot showing the effect of (a) bubble age, (b) bubble velocity, (c) melt temperature, (d) the interaction between bubble age and bubble velocity (e) the interaction between bubble age and melt temperature on the amount of hydrogen inside the bubble.

All the three process parameters as well as interactions of each of the bubble velocity and melt temperature with the bubble age were found to have a direct effect on the H diffusion into the bubble. As shown in Fig 3A, a longer holding time of the air bubble within the liquid Al, was found to boost the amount of H inside it. The same effect was observed with increasing the rotational speed of the bubble and/or the holding temperature (Fig 3B and 3C). In addition, the influence of the holding time is more significant at higher rotational speeds or holding temperatures, as could be inferred from Fig 3D and 3E.

**Fig 4:**
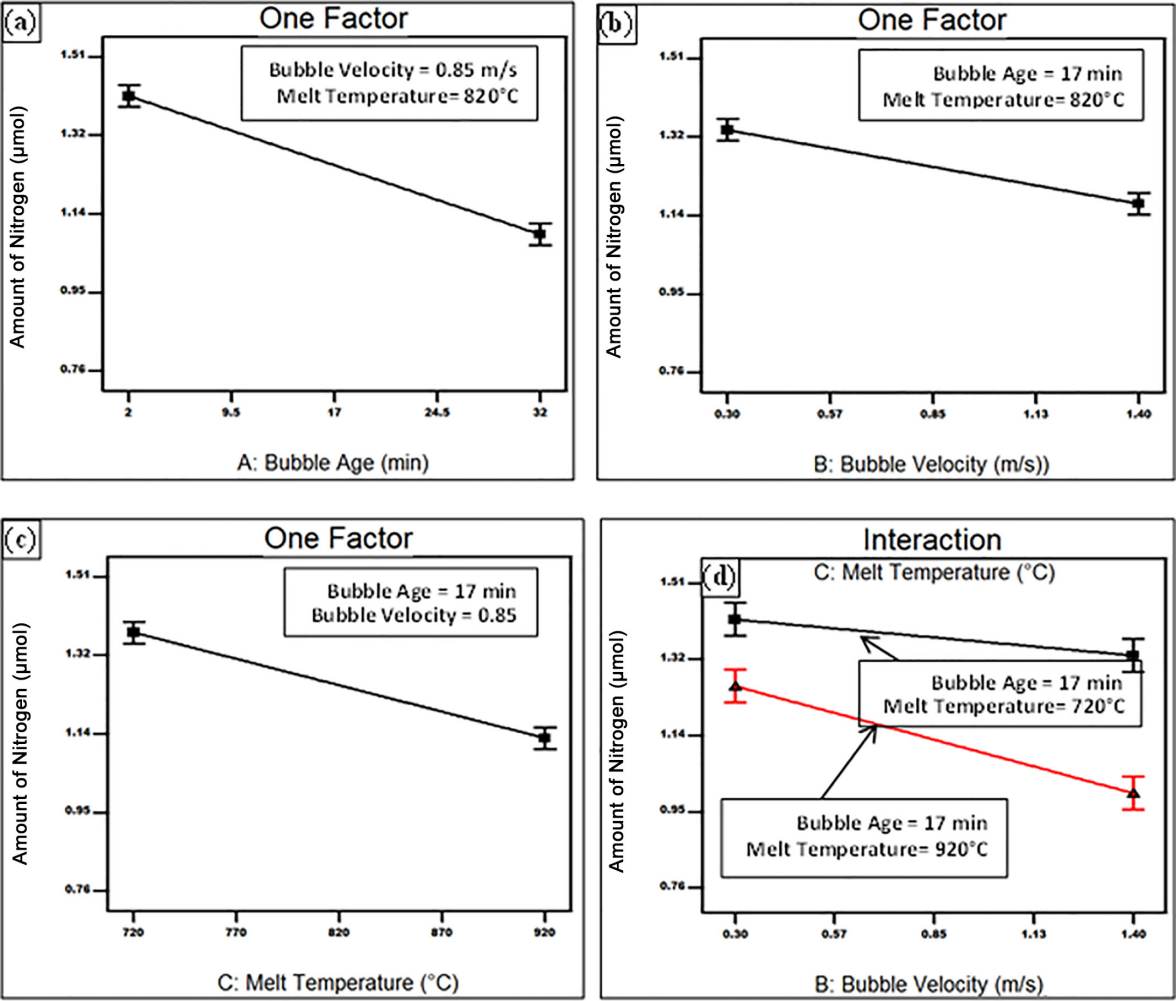
Response surface plot showing the effect of (a) bubble age, (b) bubble velocity, (c) melt temperature, (d) the interaction between bubble velocity and melt temperature on the amount of nitrogen inside the bubble.

Finally the consumption of nitrogen from inside the bubble was suggested by the model to be directly proportional to bubble age, bubble velocity and melt temperature (See Fig 4A, 4B and 4C). The interaction between bubble velocity and melt temperature was found also to significantly affect the N consumption. At the same holding time, increasing the holding temperature resulted in a steeper slop of the relationship between the bubble velocity and the amount of N inside the bubble, as seen in Fig 4D.

From Figs 2 to 4, it could be inferred that the early stages of holding were characterized by a rapid loss of oxygen leading it to reach a value of about 0.05 m in about quarter an hour. This was followed by the consumption of nitrogen and an increase in the hydrogen content at different rates, depending on the casting conditions. The rates were significantly increased upon increasing the holding time and temperature, and stirring velocity. It should be emphasized that the achieved inferences for the change of the gaseous content of the bubble are only valid within the investigated range of process parameters. Other situations, such as severe surface turbulence associated with higher stirring speeds, might take place outside the examined range, which might affect the consumption rates of the bubble atmosphere and/or the H diffusion into the bubble.

Sleppy [19] suggested that the presence of disarray in the oxide layer such as cracks or pores can create leakage paths for oxygen atoms to travel inwards through the first formed oxide layer. In this experiment, the stirring action of the air bubble in the melt (which simulated the movement of a bifilm within a flowing liquid metal) would induce a considerable stress in the oxide layer (between the melt and the bubble), which may lead to its cracking. Such cracks would allow the liquid Al to come into contact with the O and N inside the bubble resulting in the consumption of gases, at considerably higher rates than if diffusion through the oxide film was the controlling mechanism. Also, they might permit the passage of H into the bubble interior.

These results were in agreement with previous results by Raiszadeh and Griffiths [10] and El-Sayed et al. [11] of the change in the amount of gases within the bubbles held inside different Al alloy melts for varying lengths of time. The authors suggested that since the Gibbs Free Energy of formation of MgAl_2_O_4_ (-940 kJmol^-1^ O_2_ at 1000K) is significantly higher than that of AlN (-423.6 kJ mol^-1^ N_2_ at 1000K) [20,21], initially oxygen would be consumed and then nitrogen. In addition, the hydrogen content of the bubbles was increased as the melt was kept longer inside the liquid metal. It should be also emphasized that the Gibbs Free Energies of formation (for the reaction of one mole of N_2_ at 1000K) of AlN, Mg_3_N_2_ and Si_3_N_4_ were -423.6, -257.14 and -206.61 kJ mol^-1^ N_2_ respectively [20,21], indicating that AlN was the most favorable nitride to form for the 2L99 alloy composition.

The increase in the stirring velocity from 115 rpm (0.3 m/s) to 540 rpm (1.4 m/s) was shown to decrease the amount of oxygen inside the bubble and to increase the amount of hydrogen diffused into it. This might be due to the increase in the rate of cracking experienced by the oxide layer forming the interface between the bubble and the melt which increased the rate of consumption of oxygen and nitrogen and the rate of diffusion of hydrogen.

On the other hand, raising the holding temperature from 720 to 920°C was found to accelerate the reaction between the Al melt and the oxygen and nitrogen inside the bubble. This is in agreement with Sleppy [22], who reported a positive effect of the increase in temperature on the rate of oxidation of high-purity aluminium melted in a vacuum at temperatures from 660°C to 850°C and oxidised in 16 kPa dry oxygen. Also, the H content inside the bubble was significantly increased when the melt temperature was increased from 720 to 920°C. Ransley and Neufeld [23] reported an increase in H solubility in liquid Al of about 3 times when increasing the melt temperature from 700 to 900°C. Increasing the H content of the melt would increase the rate of H diffusion from the melt into the bubble, due to the increased difference in the H content.

**Fig 5:**
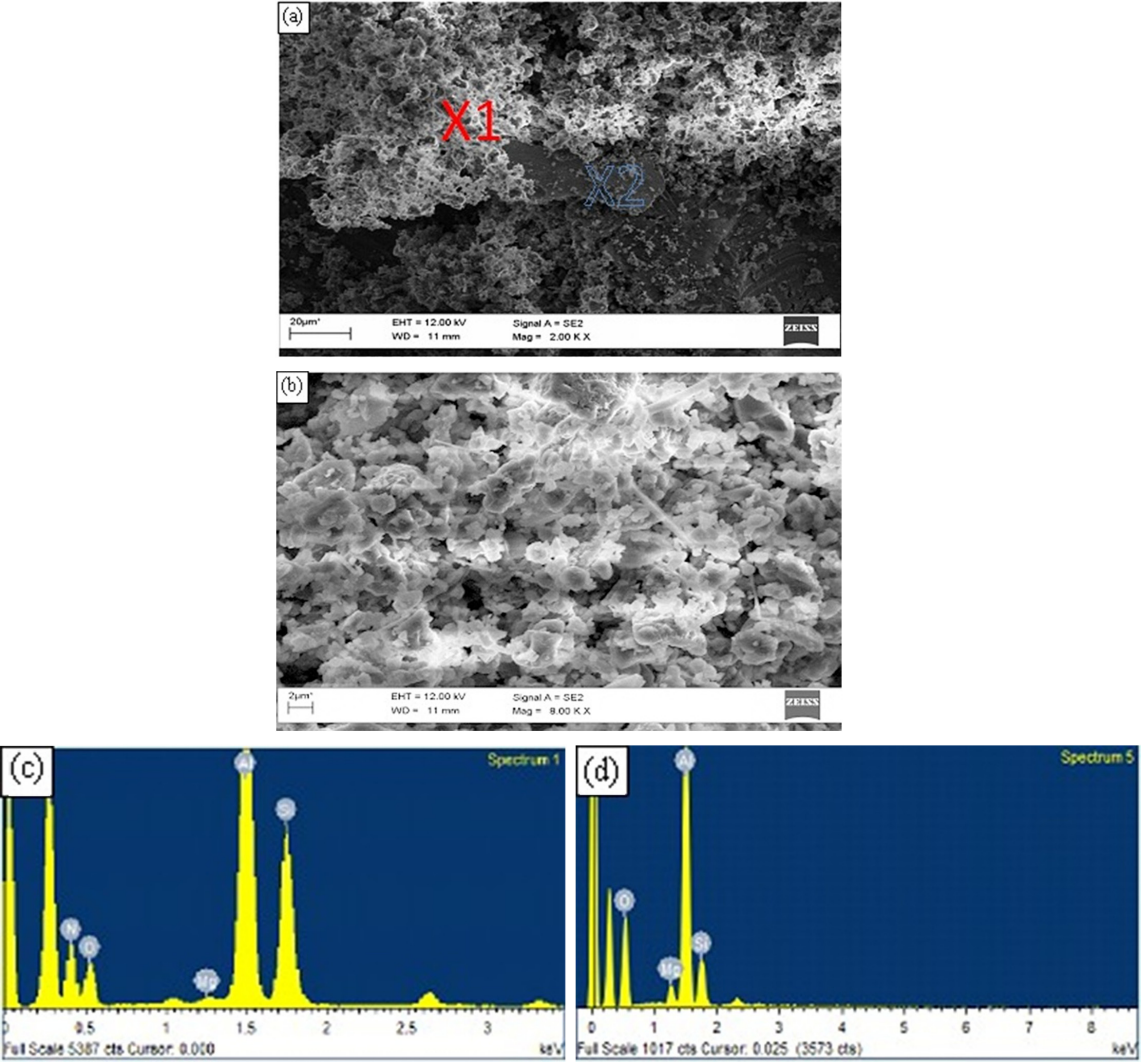
(a) SEM micrograph of a solidified sample taken from an experiment with 2L99 alloy, from inside a bubble from experiment 2, (b) A magnification of the AlN layer shown in (a), (c) and (d) EDX spectra at locations X1 and X2 in (a) respectively.

**Fig 6:**
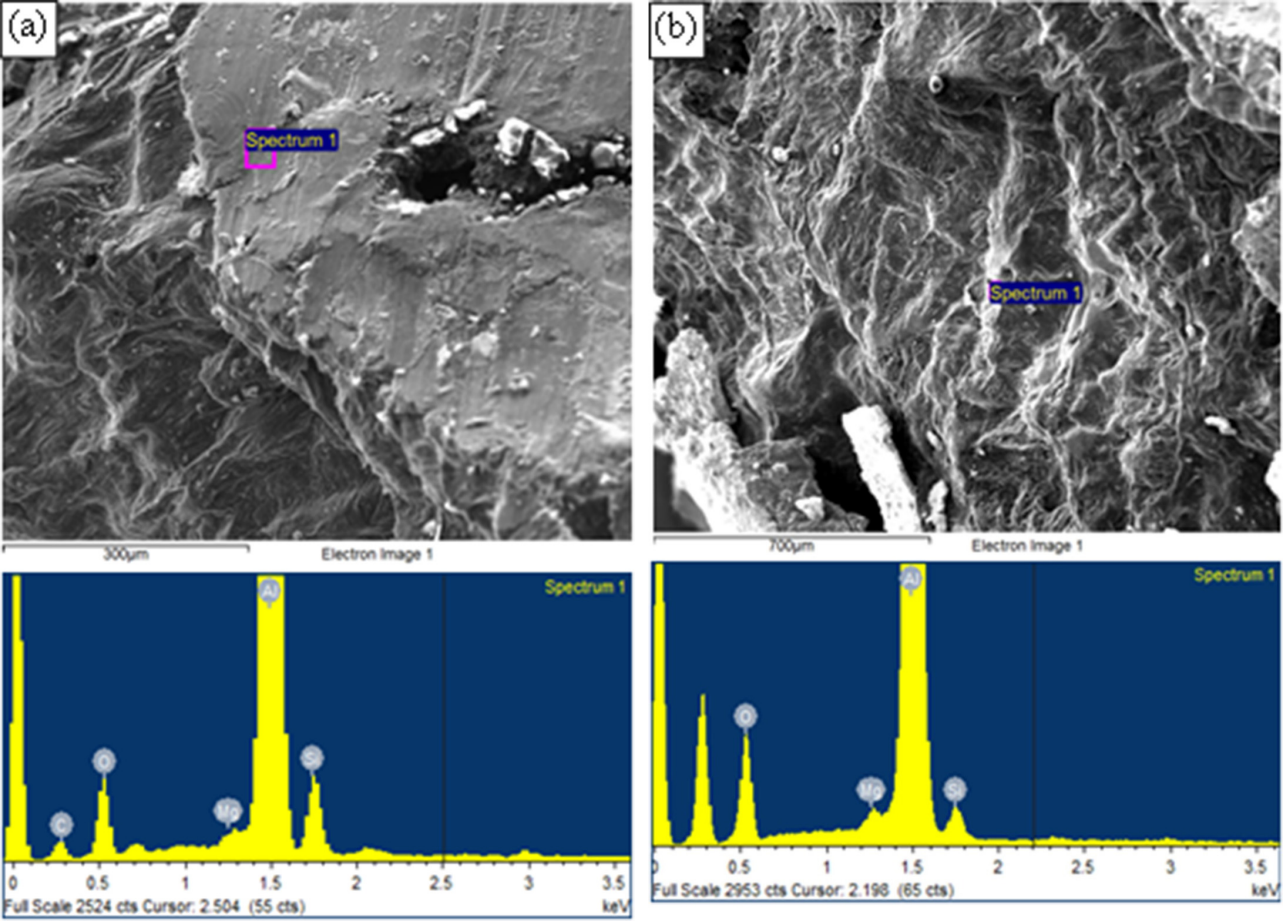
SEM images with corresponding EDX spectra for the surfaces of solidified samples taken from experiments from inside bubbles of different samples, (a) run number 7 and (b) run number 14.

Fig 5 shows an example of adjacent zones of aluminium nitride (the lighter phase) and spinel (the darker phase), found on the surfaces of a sample, taken from inside a bubble that was rotated at a speed of 540 rpm (equivalent to an angular velocity of 1.4 m/s) in a 2L99 melt at a temperature of 820°C for 17 minutes, before solidification (a sample from run number 2 of the parametric combination of the DoE shown in Table 3). Fig 5A is an SEM image illustrating two layers, one of AlN and one of MgAl_2_O_4_, while (b) shows a higher magnification view of the AlN layer. This could be an indication that oxygen and nitrogen within the trapped air bubble were consumed by the surrounding melt producing MgAl_2_O_4_ spinel and aluminium nitride. The corresponding EDX spectra, at points X1 and X2 are given in (c) and (d) respectively, confirmed the presence of MgAl_2_O_4_ and AlN on the sample surfaces. There was an oxygen peak associated with the EDX spectrum of the aluminium nitride layer, which would suggest that formation of the AlN layer occurred on a previously formed oxide layer. Also, the EDX spectra contained a peak for carbon which was suggested to be due to the contamination of the atmosphere inside the SEM. Fig 6A and 6B show SEM micrographs of the surfaces of samples taken from bubbles of sample runs 7 and 14, respectively, which showed the layered nature of the oxide films. EDX analysis on the solidified surfaces within the bubbles detected peaks for aluminium, magnesium and oxygen, suggesting the presence of spinel on these surfaces. No nitrogen peaks were detected in the EDX spectra, indicating that under the experimental conditions of stirring velocity and time, and melt temperature, no detectable AlN layers were formed on the surface of the melt in contact with the atmosphere inside the hole of the steel holder.

The results of the current research could be suggestion that the movement of a double oxide film defect within the molten metal (which has been imitated by the stirring action of the air bubble) would generate considerable stresses in the oxide skin of the defect leading to its cracking. This would allow the oxygen and nitrogen, within the atmosphere of bifilm flowing inside a 2L99 Al melt, to be consumed by reaction with the surrounding liquid metal, with initially the reaction of oxygen to form MgAl_2_O_4_ and then the reaction of nitrogen to form AlN. Also, hydrogen was suggested to consistently diffuse into the defect. The reaction rates and the rate of H diffusion were found to be significantly influenced by the holding process parameters. The rates were maximum as the stirring velocity and/or the melt temperature increased. Also, increasing the holding time was predicted to decrease the amount of O and N while increasing the amount of H inside the defect.

**Fig 7:**
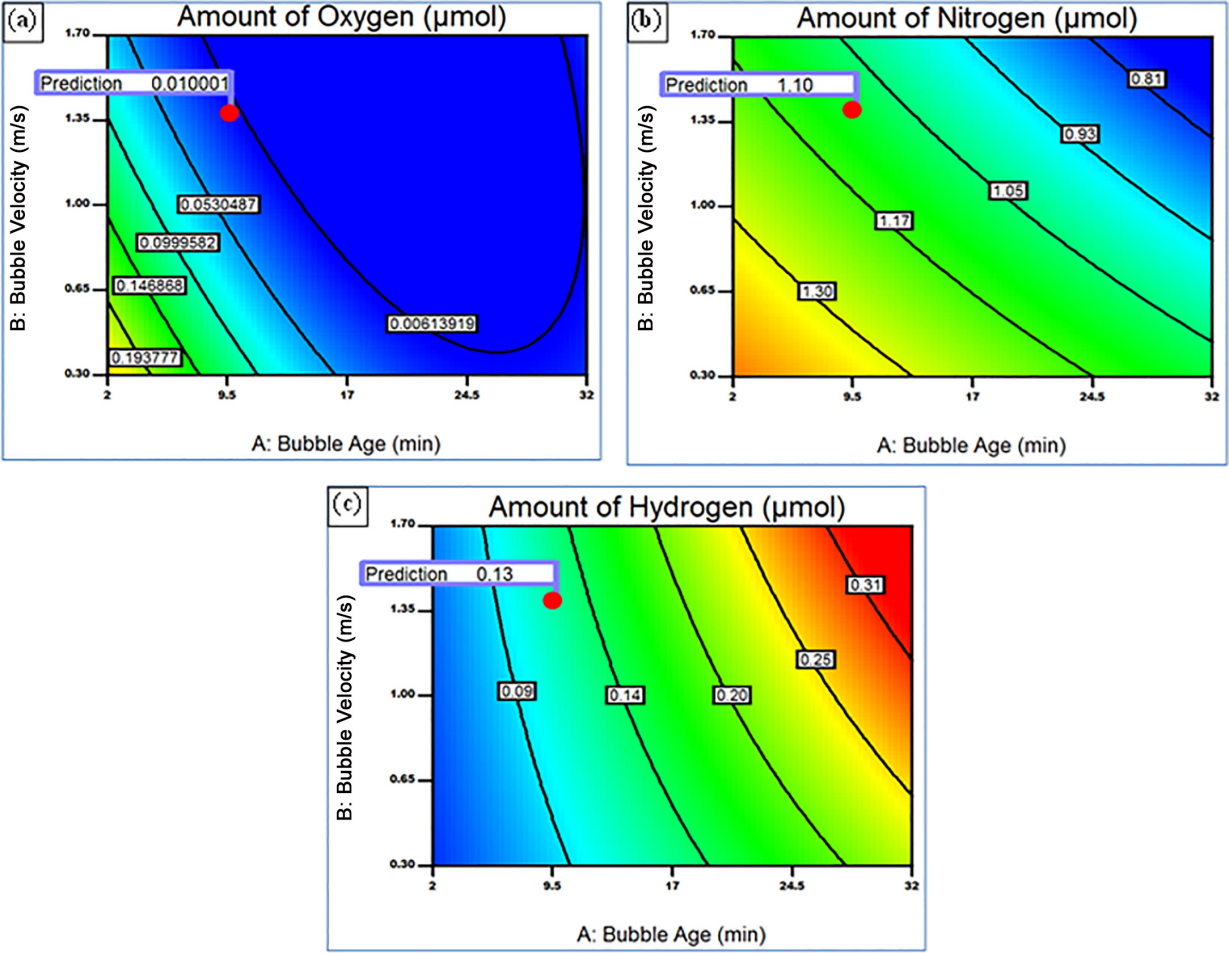
Predicted optimum holding time (bubble age), bubble velocity and melt temperature corresponding to minimum amount of gases inside the bubble (a) Oxygen, (b) Nitrogen and (c) Hydrogen.

To explore the optimum casting parameters at which the least quantity of different gases inside the bubble can be achieved, an optimisation study was carried out. The objective function was set to minimise the amount of O, N and H within the bubble. The experimental data was analysed by Design-Expert software and the genetic algorithm was used to predict the process parameters based on the objective function. The response equations describing the amount of gases in the bubble atmosphere in terms of the key process parameters (shown in Equations (2) and (3) and the related coefficients listed in Table 3) were solved simultaneously. The results by Design-Expert software are shown in Fig 7 which shows the contour plot for the optimisation function to obtain minimum amount of gases, for a range of bubble ages and velocities. The model suggested that the optimum values of holding time, stirring speed and melt temperature would be 9.7 min, 1.4 m/s and 920°C, respectively. At these values the predicted amounts of O, N and H inside the air bubble were 0.01, 1.1 and 0.13 μmol, respectively, (Fig 7A, 7B and 7C, respectively).

In their study of the effect of holding different Al alloy castings in the liquid state for different periods before solidification, El-Sayed and co-authors reported the occurrence of two competing mechanisms during the holding treatment. The first mechanism was the consumption of the atmosphere inside bifilms by reaction with the surrounding melt which would decrease the size of the defects and consequently improve the mechanical properties. The second was the diffusion of H into the bifilm’s interior, swelling them and making the properties worse. The authors showed that holding Al casting in the liquid state at 800°C for 10 minutes before solidification was associated with the best Weibull moduli of the tensile strength and elongation which could be suggestion that under these circumstances the oxide film defects might have a morphology that was least detrimental to the properties of the castings [24]. This could be a confirmation of the results of the present work as the optimisation study, performed based on experimental data, indicated that allowing an air bubble (as an analogue for a double oxide film defect) to move in a 2L99 alloy melt with a velocity of 1.4 m/s for about 10 minutes at a temperature of about 920°C could cause the bubble (or the bifilm), to lose a substantial amount of its initial internal atmospheres, while absorbing little hydrogen from the surrounding melt. The implication of this prediction is that even if not all values predicted the model might be applicable in practical applications, it is still beneficial to keep close to them as much as possible if a holding treatment is going to be applied to an Al casting before solidification.

To summarise, the results of the trapped bubble experiment suggest that double oxide films, once formed, quickly undergo changes in their internal atmosphere which might affect their size and shape. These changes are the rapid consumption of oxygen, and a slower accumulation of hydrogen. The consumption of nitrogen is also a slower process, occurring subsequent to the reaction of oxygen, although complete oxygen consumption does not appear to be required before the formation of AlN. The consumption of the bifilm atmosphere and the H diffusion into the defect were found directly related to the holding time and temperature as well as the relative velocity between the bifilm and the melt.

## Conclusions

1. The atmosphere within the trapped air bubble in a 2L99 Al melt was consumed by reaction with the surrounding liquid metal. First oxygen was consumed to produce MgAl_2_O_4_, then nitrogen reacted to form AlN.
2. Hydrogen was found to diffuse into the bubble, and hence would diffuse into a double oxide film defect.
3. The rates of reaction of oxygen and nitrogen within the internal atmosphere of a bubble, and the rate of hydrogen diffusion into it were found to be significantly affected by the parameters of the holding process. The rates increased by increasing the holding time, bubble velocity and/or melt temperature.
4. It was shown that the amount of different gases inside a bubble (or a bifilm) can be minimised using an optimum set of the holding process parameters predicted using a design-of-experiments approach. This might lead to a defect being least harmful to the mechanical properties of the Al castings.

## Supporting Information

S1 File A complete Design Expert file names "Design of Experiment" containing the design data, the analysis of different amount of gases as well as the optimization performed. (DX7)

S1 Table Matrix building parameters and the amount of different gases within the bubbles. (DOCX)

S2 Table Response surface model coefficients for the amount of oxygen, nitrogen and hydrogen inside the bubble. (DOCX)
