# Supplementary material for: The Behaviour of Bifilm Defects in Cast Al-7Si-Mg Alloy
Source: PLoS One. 2016 Aug 16;11(8):e0160633. doi: 10.1371/journal.pone.0160633 (PMC4987044; doi:10.1371/journal.pone.0160633)
Supplement: S1 Table — (DOCX) [file pone.0160633.s002.docx]

S1 Table Matrix building parameters and the amount of different gases within the bubbles.

| Run | Bubble Age (minute) | Bubble Velocity (ms^-1^) | Melt Temperature (°C) | Amount of Oxygen (µmol) | Amount of Nitrogen (µmol) | Amount of Hydrogen (µmol) |
| --- | --- | --- | --- | --- | --- | --- |
| 1 | 32 | 0.85 | 820 | 0.01 | 1.08 | 0.25 |
| 2 | 17 | 1.4 | 820 | 0.01 | 1.18 | 0.17 |
| 3 | 17 | 0.85 | 820 | 0.01 | 1.25 | 0.15 |
| 4 | 2 | 1.4 | 920 | 0.08 | 1.19 | 0.05 |
| 5 | 32 | 0.3 | 720 | 0.01 | 1.3 | 0.13 |
| 6 | 32 | 0.3 | 920 | 0.01 | 1.05 | 0.22 |
| 7 | 2 | 0.3 | 720 | 0.3 | 1.51 | 0.02 |
| 8 | 32 | 1.4 | 720 | 0.01 | 1.15 | 0.23 |
| 9 | 2 | 0.3 | 920 | 0.25 | 1.4 | 0.03 |
| 10 | 2 | 0.85 | 820 | 0.19 | 1.38 | 0.03 |
| 11 | 17 | 0.85 | 820 | 0.01 | 1.23 | 0.16 |
| 12 | 17 | 0.85 | 720 | 0.02 | 1.39 | 0.12 |
| 13 | 17 | 0.85 | 820 | 0.01 | 1.27 | 0.14 |
| 14 | 2 | 1.4 | 720 | 0.2 | 1.51 | 0.02 |
| 15 | 32 | 1.4 | 920 | 0.01 | 0.76 | 0.32 |
| 16 | 17 | 0.3 | 820 | 0.04 | 1.41 | 0.1 |
| 17 | 17 | 0.85 | 920 | 0.02 | 1.2 | 0.16 |
