# Supplementary material for: The Behaviour of Bifilm Defects in Cast Al-7Si-Mg Alloy
Source: PLoS One. 2016 Aug 16;11(8):e0160633. doi: 10.1371/journal.pone.0160633 (PMC4987044; doi:10.1371/journal.pone.0160633)
Supplement: S2 Table — (DOCX) [file pone.0160633.s003.docx]

S2 Table Response surface model coefficients for the amount of oxygen, nitrogen and hydrogen inside the bubble.

| Coefficient | Model for the Amount of Hydrogen | Model for the Amount of Nitrogen | Model for the Amount of Oxygen |
| --- | --- | --- | --- |
| b_o_ | 0.14 | 1.25 | 0.014 |
| b_1_ | 0.100 | -0.16 | -0.097 |
| b_2_ | 0.029 | -0.088 | -0.030 |
| b_3_ | 0.026 | -0.13 | -0.017 |
| b_4_ | 0.022 | -0.029 | 0.034 |
| b_5_ | 0.018 | -0.026 | 0.021 |
| b_6_ | 2.500E-003 | -0.044 | -8.750E-003 |
| b_7_ | 0 | 0 | 0.084 |
| b_8_ | 0 | 0 | 8.592E-003 |
| b_9_ | 0 | 0 | 3.592E-003 |
